# Immunopathological Features of Canine Myocarditis Associated with* Leishmania infantum* Infection

**DOI:** 10.1155/2016/8016186

**Published:** 2016-06-19

**Authors:** Alessandro Costagliola, Giuseppe Piegari, Iwona Otrocka-Domagala, Davide Ciccarelli, Valentina Iovane, Gaetano Oliva, Valeria Russo, Laura Rinaldi, Serenella Papparella, Orlando Paciello

**Affiliations:** ^1^Unit of Pathology, Department of Veterinary Medicine and Animal Productions, University of Naples Federico II, 80137 Naples, Italy; ^2^Department of Pathological Anatomy, Faculty of Veterinary Medicine, Warmia and Mazury University in Olsztyn, 10-701 Olsztyn, Poland; ^3^Unit of Internal Medicine, Department of Veterinary Medicine and Animal Productions, University of Naples Federico II, 80137 Naples, Italy; ^4^Unit of Parasitology, Department of Veterinary Medicine and Animal Productions, University of Naples Federico II, 80137 Naples, Italy

## Abstract

Myocarditis associated with infectious diseases may occur in dogs, including those caused by the protozoa* Neospora caninum*,* Trypanosoma cruzi*,* Babesia canis,* and* Hepatozoon canis*. However, although cardiac disease due to* Leishmania* infection has also been documented, the immunopathological features of myocarditis have not been reported so far. The aim of this study was to examine the types of cellular infiltrates and expression of MHC classes I and II in myocardial samples obtained at necropsy from 15 dogs with an established intravitam diagnosis of visceral leishmaniasis. Pathological features of myocardium were characterized by hyaline degeneration of cardiomyocytes, necrosis, and infiltration of mononuclear inflammatory cells consisting of lymphocytes and macrophages, sometimes with perivascular pattern; fibrosis was also present in various degrees. Immunophenotyping of inflammatory cells was performed by immunohistochemistry on cryostat sections obtained from the heart of the infected dogs. The predominant leukocyte population was CD8+ with a fewer number of CD4+ cells. Many cardiomyocytes expressed MHC classes I and II on the sarcolemma.* Leishmania* amastigote forms were not detected within macrophages or any other cell of the examined samples. Our study provided evidence that myocarditis in canine visceral leishmaniasis might be related to immunological alterations associated with* Leishmania* infection.

## 1. Introduction 

Canine leishmaniasis is a zoonotic disease caused by the protozoan parasite* Leishmania* spp. [1]. The disease has a worldwide distribution and is considered endemic in more than 70 countries, mainly distributed in Africa, Asia, Latin America, and Mediterranean regions [1, 2]. The domestic dog is confirmed to be the most important reservoir of human infection; in the Mediterranean region and New World, the one responsible for canine visceral leishmaniasis (CVL) in dogs is* Leishmania infantum* (syn:* L. chagasi* in the New World) [3, 4]. Even if congenital and sexual transmission have been demonstrated, the main route of transmission of the parasite among dogs, and from dogs to humans, is the bite of infected female phlebotomine sandflies [5, 6]. CVL is a multisystemic disease usually characterized by chronic progression with cutaneous and visceral clinical signs which become more and more evident as the infection progresses [7]. Enlargement of lymph nodes, dermal and ocular lesions, splenomegaly, pale mucous membrane, and weight loss are the main clinical findings [8, 9], whereas the most common laboratory abnormalities are hypoalbuminemia, hyperglobulinemia, anemia, azotemia, and proteinuria [10, 11].

Canine myocarditis is a rarely diagnosed disease which can be caused by noninfectious (e.g., autoimmune reactions, toxins, trauma, and heat stroke) or infectious agents such as bacteria (i.e.,* Staphylococcus*,* Streptococcus*,* Citrobacter*,* Bartonella*, and* Borrelia*), viruses (e.g., parvovirus), fungi (i.e.,* Coccidioides*,* Cryptococcus*, and* Aspergillus*), and protozoa (*Leishmania*,* Toxoplasma*,* Hepatozoon*, and* Babesia*) [12–16]. Depending on the aetiology, myocarditis can have various histopathologic patterns. It is usually nonspecific and, although it is stated in the histopathologic examination, its direct cause can rarely be determined as discussed by Janus et al. [12]. The aim of this study is to evaluate the phenotype of inflammatory cells and define the immunopathological features of myocarditis associated with* L. infantum* infection in dogs.

## 2. Materials and Methods 

### 2.1. Animals and Sampling

Fifteen crossbreed dogs, 8 males and 7 females, aged 7 to 11 years, living in an endemic area for* Leishmania* in southern Italy, were selected for the study. All dogs had an established intravitam diagnosis of leishmaniasis by serological and parasitological methods [17]. Moreover, laboratory abnormalities and clinical signs characteristic of visceral leishmaniasis were also found in all dogs.

The dogs were serologically negative for the main infectious agents responsible of myocarditis (*Ehrlichia canis, Toxoplasma gondii, Babesia canis, Rickettsia rickettsii, Leptospira, Borrelia burgdorferi,* and* Neospora caninum*) and did not show any other clinical signs of heart failure.

As a control, 3 crossbreed dogs, 2 males and 1 female, aged 7 to 11 years, living in the same endemic area of southern Italy, without clinical or laboratory evidence of leishmaniasis were used in the study; these dogs were serologically and parasitologically negative for* L. infantum* infection. Each animal used in the study died naturally or was humanely euthanized due to severe clinical conditions and poor prognosis and underwent full necropsy which confirmed the absence of concomitant diseases. Control group dogs died because of road accident trauma and underwent full necropsy which excluded the presence of any infectious or noninfectious disease.

At necropsy, specimens of myocardium, about 1 cm × 1 cm × 1 cm (L × W × H), were collected from the right atrium, ventricular free walls, and the interventricular septum as described by Rosa et al. [18]. Samples were frozen in isopentane precooled in liquid nitrogen and stored at −80°C.

### 2.2. Histopathology and Immunohistochemistry

Cryostat sections (5 *μ*m thick) were stained with hematoxylin and eosin (H&E) for histopathological examination to assess a definitive diagnosis of myocarditis.

Immunohistochemical examination was carried out as previously described [19]. In brief, frozen myocardial specimens were sectioned (5 *μ*m thick), dried at room temperature for 1 hour, fixed in acetone at 4°C for 5 minutes, and then blocked for endogenous peroxidase in 0.3% H_2_O_2_ in methanol solution for 20 minutes. Sections were incubated overnight at 4°C with primary antibodies against canine leukocyte antigens diluted in 0.01 M phosphate-buffered saline (PBS), pH 7.2–7.4, as follows:

- CD3 (mouse monoclonal antibody against canine CD3, T lymphocytes: from P. Moore, UC Davis) diluted 1 : 50.
- CD4 (mouse monoclonal antibody against canine CD4, MHC class II-restricted cells, T, tissue macrophages: from P. Moore, UC Davis) diluted 1 : 50.
- CD8*α*, CD8*β* (mouse monoclonal antibody against canine CD8*α*, CD8*β*, MHC class I- restricted cells; cytotoxic T lymphocytes: from P. Moore, UC Davis) diluted 1 : 50.
- CD79*α* (mouse monoclonal mouse anti-human CD79*α*, B-linage cells clone HM57, DAKO A/S, Denmark) diluted 1 : 50.
- MHC I (mouse monoclonal antibody against MHC class I, clone H58A: from VMRD, Inc., USA) diluted 1 : 100.
- MHC II (mouse monoclonal antibody against MHC class II, clone H34A, from VMRD, Inc., USA) diluted 1 : 50.

Slides were washed with PBS, then incubated with biotinylated secondary antibody, and labeled with streptavidin biotin for 30 minutes at room temperature, followed by incubation with streptavidin conjugated to horseradish peroxidase (LSAB Kit, DakoCytomation, Denmark). The reaction was revealed by diaminobenzidine treatment (DakoCytomation, Denmark) and finally, sections were counterstained with Mayer's haematoxylin. In the negative control sections, the primary antibody was either omitted or replaced with normal serum.

Approximately 20 fields at 20x magnification were evaluated for each section by two independent pathologists (AC, OP) with a concordance rate of 97%.

The inflammatory cell immunoreactions were scored as follows:

 0 (not detected).
 1 (percentage of immunoreactive inflammatory cells per section 1–25%).
 2 (percentage of immunoreactive inflammatory cells per section 26–50%).
 3 (percentage of immunoreactive inflammatory cells per section >50%).

### 2.3. Statistical Analysis

The relationship between the different types of infiltrating immune cells and MHC class I expression was evaluated using Spearman's Rho correlation (Past 3.10 software).

### 2.4. Ethics Statement

Necropsies were performed for diagnostic purposes after receiving the consent of the owner. Each owner consented to the use of tissues for research purposes, according to the internal rules of the Diagnostic Service of Pathology and Animal Health of the University of Naples Federico II. All the procedures were performed for diagnostic purpose; thus the study did not require any consent or ethical approval according to the European Directive 2010/63/EU.

## 3. Results

### 3.1. Noninfected Control Dogs

Myocardial samples from healthy control dogs, serologically and parasitologically negative for* L. infantum*, showed neither morphologic alterations nor inflammatory cells infiltrates. MHC classes I and II were expressed by endothelial cells of arterioles, venules, and capillaries.

### 3.2. *Leishmania infantum* Infected Dogs

**Figure 1:**
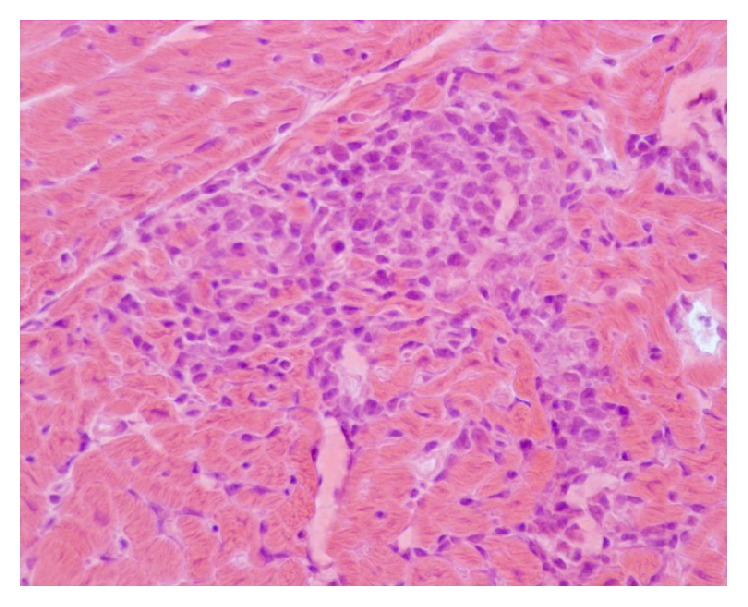
Myocardium, histopathological findings of a dog infected by* L. infantum*. Severe interstitial infiltration of mononuclear cells. H&E original magnification 40x.

At H&E stain all affected dogs (100% of studied cases) showed variable numbers of mononuclear cells, represented by lymphocytes and some macrophages, sometimes in a perivascular pattern (35.7%, 5/14). In some cases (14.3%, 2/14) a nonsuppurative granulomatous myocarditis, characterized by severe interstitial infiltration of mononuclear cells, was identified (Figure 1). Inflammatory cells infiltration was present in 71.4% (10/14) of the cases with cardiomyocytes hyaline degeneration and necrosis. Furthermore, we observed fibrosis in 9/14 (64.3%) cases; in 5 out of 9 cases (55.5%) fibrosis was mild and in other 4 cases (44.4%) fibrosis was moderate. In none of the studied cases* L. infantum* amastigotes were detected within macrophages.

**Figure 2:**
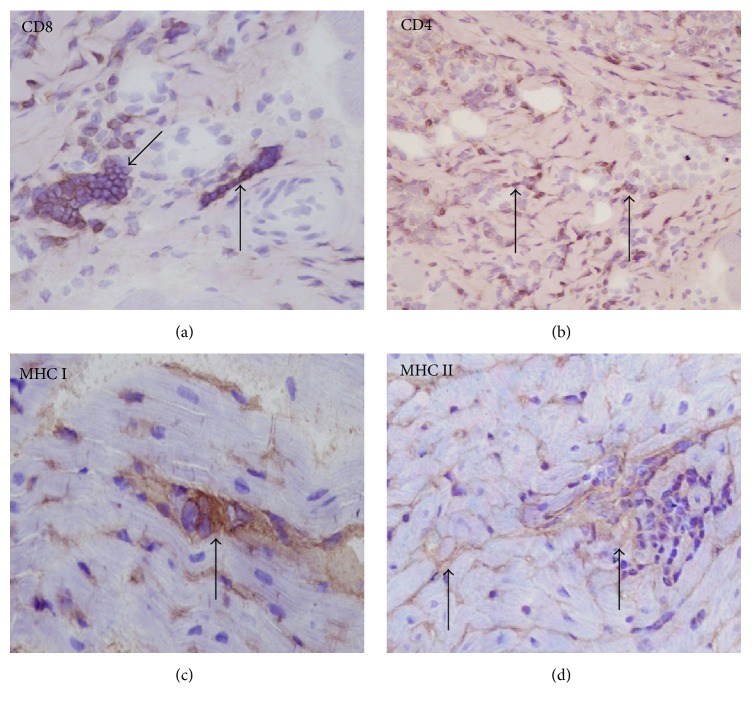
Myocardium, histopathological findings in a dog infected by* L. infantum*: (a) CD8 immunoperoxidase stain showing CD8+ T cells (arrows). (b) CD4 immunoperoxidase stain showing CD4+ T cells (arrows). (c) MHC class I immunoperoxidase stain showing abnormal positivity within cardiomyocytes (arrow). (d) MHC class II immunoperoxidase stain showing abnormal positivity within cardiomyocytes (arrows) (immunohistochemistry, HRP method, original magnification 40x).

Inflammatory cells phenotype was identified based on staining pattern of monoclonal antibodies against cell surface proteins. In all cases, independently of the severity and the pattern of inflammation, the predominant cell populations were CD3, CD8, and CD4 positive with predominance of CD8+ T cells (Figure 2(a)) compared to CD4+ cells (Figure 2(b)). Only in few cases were a small number of CD79*α*+ cells rarely detected within the inflammatory infiltrates. Vascular adventitia, endothelial cells, and cellular infiltrates within the myocardium stained intensely for MHC classes I and MHC class II antigens. In addition, many cardiomyocytes had MHC class I (Figure 2(c)) and class II positivity on the sarcolemma (Figure 2(d)).

A positive relationship was observed between the CD8 positive cells and MHC class I expression (*ρ* = 0.854; *P* < 0.05).

Results of the immunohistochemistry are summarized in Table 1.

## 4. Discussion 

Canine myocarditis associated with* L. infantum* infection in dogs has been already described [20, 21]; however, the inflammatory pattern and its immunopathological features have never been fully investigated so far.

Pathological changes of myocardium observed in our cases including degeneration and necrosis of cardiomyocytes and interstitial infiltration of mononuclear inflammatory cells represented by macrophages and lymphocytes confirmed findings of previously published reports [15, 18]. In none of myocardial samples* Leishmania* amastigotes were detected, and this aspect seems to be in accordance with the report of Alves et al. [16].

The predominant inflammatory infiltrate cell types were CD8+ T lymphocytes and macrophages; macrophages were distinguished from lymphocytes, at light microscopy, by morphological features and they were MCH immunoreactives. However, CD4+ T cells were also found. It is now well accepted that the progression of* L. infantum* infection in dogs, notably the worsening or the regression of clinical signs, is the result of a multifactorial and complex interaction among the virulence of parasite, the environment (e.g., repeated bites by infected vectors), and the immune response of the host [22, 23]. The latter seems to play a key role, as* L. infantum* induces a mixed Th1 and Th2 response in CVL and the control of parasite replication, disease progression, or cure are strictly associated with the balance of these two patterns of immune system reaction [22]. The protective immunity against the parasite is mediated by CD4+ Th1 lymphocytes which release cytokines (*γ*-interferon, IL-2, and TNF-*α*) promoting macrophage anti-*Leishmania* activity through nitric oxide production that is responsible for parasite killing by apoptosis [24, 25]; moreover, macrophages infected by* Leishmania* amastigotes may also be lysed by CD8+ cytotoxic T lymphocytes even if this mechanism may be suppressed by the presence of high parasitic load [26, 27]. Contrarily, the Th2 humoral immune response, involving an increase of B cells and plasma-cells activity, is not protective and is associated with hyperglobulinemia and generation of autoantibodies, antihistone antibodies, and circulating immune complexes responsible for inflammation in almost every organ and tissue (e.g., glomerulonephritis, vasculitis, uveitis, polyarthritis, and myositis) [28–32]. The presence of both CD4+ and CD8+ inflammatory cells found in our study suggests that the dog immune system responds with a Th1/Th2 mixed response to* L. infantum* infection and this mechanism could be at the basis of myocardial injury and it was already demonstrated in canine inflammatory myopathy associated with* L. infantum* infection [19].

Detection of MHC classes I and II expression in cardiomyocytes was a common finding in the majority of samples from infected dogs [19]. Immunohistochemical detection of sarcolemmal MHC classes I and II is considered as a valid test for immune-mediated idiopathic inflammatory myositis in humans and dogs, in presence or absence of inflammatory cells infiltration [19]. Notably, MHC I and MHC II expression has been correlated to the active role of muscle fibers in antigen presentation and in initiating and maintaining pathological events in immune-mediated myositis [33–36]. CD8/MHC-I complex has already been described in other infectious and immune-mediated myositis of humans and dogs [19, 37, 38]. In dog the most common immune-mediated myopathies are masticatory muscles myositis, polymyositis, and dermatomyositis [39]. In 2009, Paciello et al. reported an immune-mediated inflammatory myopathy associated with* L. infantum* infection [19]. Our data suggest that at least one pathologic mechanism resulting in myocardial inflammation in CVL can be an immune-mediated pathway as previously described in* L. infantum* infection associated myositis in dogs [28]. Furthermore, supporting this hypothesis, many parasites and viruses have been proposed as responsible factors of systemic diseases resulting in immune-mediated inflammatory myopathies in both humans and dogs [33, 34, 37].

## 5. Conclusion 

Our data provided an initial antigenic characterization of infiltrating mononuclear cells and MHC classes I and II expression in myocarditis associated with* L. infantum* infection in dog. Our study provides evidence that during leishmaniasis myocarditis can occur with morphological and immunophenotypical pattern superimposable to canine myositis associated with* L. infantum *infection. Finally, our results, if confirmed on larger scale, could be used to improve therapeutic protocols for the management of dogs affected by leishmaniasis or to address the research towards new drugs useful to modulate immune-system response in order to reduce myocardial inflammation.

## Figures and Tables

**Table 1:** Immunohistochemical results: scoring of inflammatory cells immunoreactions and MHC classes I and II expression.

Dog #	CD3+	CD4+	CD8(*α*, *β*)+	CD79+	MHC I	MHC II
1	2	0	2	0	2	2
2	3	2	3	0	3	2
3	1	0	1	0	1	1
4	2	2	3	1	3	2
5	3	1	2	0	2	1
6	1	1	1	0	1	1
7	2	0	2	0	2	2
8	3	1	3	1	3	3
9	2	2	2	0	1	2
10	1	1	1	0	1	1
11	2	1	1	0	2	2
12	1	1	1	0	1	1
13	3	1	3	0	3	3
14	2	2	2	0	1	2
15	3	2	3	1	3	2

*Scoring system applied for inflammatory cells immunoreactions*: 0 (not detected); 1 (percentage of immunoreactive inflammatory cells per section 1–25%); 2 (percentage of immunoreactive inflammatory cells per section 26–50%); 3 (percentage of immunoreactive inflammatory cells per section >50%).

*Scoring system applied for MHC classes I and II expression*: 0: absent; 1: mild; 2: moderate; 3: intense.
